# Reconstitution of Human Necrosome Interactions in *Saccharomyces cerevisiae*

**DOI:** 10.3390/biom11020153

**Published:** 2021-01-25

**Authors:** Y. Ji, L. A. Ward, C. J. Hawkins

**Affiliations:** Department of Biochemistry and Genetics, La Trobe Institute for Molecular Science, La Trobe University, Bundoora, VIC 3083, Australia; Y.Ji@latrobe.edu.au (Y.J.); L.Ward@latrobe.edu.au (L.A.W.)

**Keywords:** necroptosis, *S. cerevisiae*, kinase, phosphorylation, RIP, RHIM, necroptotic inhibitor, subcellular localization, overexpression, yeast

## Abstract

The necrosome is a large-molecular-weight complex in which the terminal effector of the necroptotic pathway, Mixed Lineage Kinase Domain-Like protein (MLKL), is activated to induce necroptotic cell death. The precise mechanism of MLKL activation by the upstream kinase, Receptor Interacting Serine/Threonine Protein Kinase 3 (RIPK3) and the role of Receptor Interacting Serine/Threonine Protein Kinase 1 (RIPK1) in mediating MLKL activation remain incompletely understood. Here, we reconstituted human necrosome interactions in yeast by inducible expression of these necrosome effectors. Functional interactions were reflected by the detection of phosphorylated MLKL, plasma membrane permeabilization, and reduced proliferative potential. Following overexpression of human necrosome effectors in yeast, MLKL aggregated in the periphery of the cell, permeabilized the plasma membrane and compromised clonogenic potential. RIPK1 had little impact on RIPK3/MLKL-mediated yeast lethality; however, it exacerbated the toxicity provoked by co-expression of MLKL with a RIPK3 variant bearing a mutated RHIM-domain. Small molecule necroptotic inhibitors necrostatin-1 and TC13172, and viral inhibitors M45 (residues 1–90) and BAV_Rmil, abated the yeast toxicity triggered by the reconstituted necrosome. This yeast model provides a convenient tool to study necrosome protein interactions and to screen for and characterize potential necroptotic inhibitors.

## 1. Introduction

Necroptosis is an inflammatory form of programmed cell death characterized by necrotic morphological features, which has been observed in multiple human diseases [1,2]. Necroptosis can be triggered by death receptors and pattern recognition receptors, such as Tumor Necrosis Factor receptor 1 (TNFR1), Toll-Like Receptor 3/4 (TLR3/4) and Z-DNA-Binding Protein 1 (ZBP1), leading to the formation of a large-molecular-weight complex, the necrosome [3,4]. Briefly, necrosome assembly is initiated by RIP Homotypic Interaction Motif (RHIM)-mediated activation of Receptor-Interacting Serine/Threonine Protein Kinase 3 (RIPK3) via oligomerization [5,6]. Active RIPK3 phosphorylates and activates Mixed-Lineage Kinase Domain-Like Protein (MLKL) at T357/S358 in its Pseudokinase Domain (PsKD) to trigger oligomerization and membrane localization of MLKL and subsequent membrane rupture [6,7,8].

MLKL is composed of an N-terminal four-helix bundle (4HB) domain and a C-terminal PsKD domain connected by a two-helix linker [9]. When inactive, the 4HB domain is buried and inhibited by the C-terminal two-helix linker and PsDK domain [10,11]. When exposed, the 4HB domain contains a positively charged interface that forms electrostatic interactions with phospholipids of the plasma membrane [12]. This exposure occurs following activation of the PsKD, which triggers MLKL to oligomerize and orients the positively charged interface of the 4HB domain such that it can engage with the plasma membrane [11,13]. The mechanism by which necroptosis is stimulated by the necrosome complex is still debated. Some observations raise questions regarding the role of phosphorylation in MLKL activation. Murine MLKL can be activated by a phosphomimetic mutation (S345D) independent of RIPK3; however, the effects of orthologous phosphomimetic mutations to human MLKL (T357E/S358D or T357E/S358E) seem to be cell-line-dependent [9,14]. While ZBP1 activation induces necrosome assembly by directly binding with RIPK3 in an RHIM-dependent manner [7], necroptosis induced by ligand binding to TNF receptors and/or TLR3/4 in particular cell lines relies on the activity of Receptor-Interacting Serine/Threonine Protein Kinase 1 (RIPK1) [15]. RIPK1 can play a pro-apoptotic role [16,17], but when the apoptosis is inhibited, RIPK1 recruits RIPK3 via a RHIM-mediated interaction, initiating the assembly of the necrosome [18]. However, knockout of RIPK1 has been shown to result in RIPK3-MLKL-mediated perinatal lethality in mice [19], suggesting that RIPK1 can inhibit necroptosis in some circumstances. Therefore, the effect of the RIPK1-RIPK3 hetero-oligomeric complex on necroptosis is not fully defined. 

Once activated, MLKL oligomerizes within the necrosome, then dissociates from RIPK3 and translocates to the plasma membrane, where MLKL oligomers disrupt membrane permeability to kill cells [20,21,22]. MLKL has been identified to provoke necroptosis via distinct processes. MLKL was observed to form cation channels, resulting in cation influx and hyperpolarization of the plasma membrane [23]. Fluorescence microscopy showed MLKL aggregation in the membrane, suggesting it disrupts membrane integrity by changing the membrane composition [13]. MLKL has been identified to oligomerize into pro-necroptotic tetramers and higher-order polymers [20,24]. 

Modeling mammalian pathways in yeast has long been used to investigate the activities of mammalian proteins in a relatively naïve eukaryotic environment. Necroptotic effectors intersect with other cellular pathways in mammalian cells [25,26,27,28]; for example, in addition to activating MLKL, RIPK3 can also promote caspase-8-dependent apoptosis [29], suppress mTOR signaling [30] and activate IL-1β [31]. RIPK1 promotes caspase-8-mediated apoptosis during embryonic development [26] and induces NF-kB activation when ubiquitinated [32,33]. Hence, reconstitution of necroptotic pathway in yeast enables a specific focus on the necrosome, minimizing the potential interference of additional confounding interactions. In this study, we established a yeast model to investigate interactions between necroptotic effectors. We characterized the impact of co-expressing necrosome components on *Saccharomyces cerevisiae* viability, finding that activation of MLKL by RIPK3 kills yeast. To explore the determinants of this yeast lethality and the impact of RIPK1 on this process, we compared the effects of expression of wild-type and mutated necrosome components. We compared the proliferation of necrosome-expressing yeast in the presence or absence of small molecule or viral necroptotic inhibitors. In addition, we visualized MLKL subcellular localization in the yeast using confocal microscopy. 

## 2. Material and Methods

### 2.1. Yeast Strains and Plasmids

The yeast strain W303α was used in this study. The pGALL-(*LEU2*), pGALL-(*URA3*), pGALL-(*TRP1*), pGALL-(*HIS3*), and pGALL-(*HIS3*)-Caspase-8 plasmids used in this study have been previously reported [34,35,36]. 

GenScript (Piscataway, NJ, USA) generated synthetic genes (codon-optimized for expression in yeast) encoding full-length murine MLKL (NP_001297542.1), murine RIPK3 (NP_064339.2) or murine RIPK1 (AAH58162.1). These were subcloned between the BamHI and XbaI sites of pUC57. Those inserts were digested with BamHI and XbaI and ligated into pGALL-(*LEU2*) for yeast expression.

pGALL-(*LEU2*)-hMLKL was made by amplifying human MLKL with primers #1 and #2, amplifying pGALL-(*LEU2*) with primers #3 and #4 and linking these using Gibson Assembly^®^ Cloning Kit following manufacturer’s instructions (New England Biolabs; Ipswich, MA, USA). GenScript generated a synthetic gene (codon-optimized for expression in yeast) encoding full-length human RIPK3 (NP_006862) between the BamHI and XbaI sites of pUC57, and the insert was excised with BamHI and XbaI and ligated into pGALL-(*URA3*) for yeast expression. pGALL-(*TRP1*)-hRIPK1 was made by amplifying human RIPK1 with primers #2014 and #2015, digesting the product with EcoRI and XhoI and ligating into pGALL-(*TRP1*) cut with BamHI and XbaI.

pGALL-(*URA3*) plasmids encoding hRIPK3 mutants were made by Combined Overlap Extension Polymerase Chain Reaction (COE-PCR) [37]. Pairs of primary PCR reactions were performed using the sets of primers specified below and pGALL-(*URA3*)-hRIPK3 as a template. Then, for each construct, a mixture of 5′ and 3′ products acted as a template for a secondary PCR reaction using vector primers #1864 and #1776. These products were cut with BamHI and XbaI and ligated into BamHI/XbaI-cut pGALL-(*URA3*). The primers used for primary reactions were:

pGALL-(*URA3*)-hRIPK3^D142N^: #1864/#1966 and #1967/#1776
pGALL-(*URA3*)-hRIPK3^S227A^: #1864/#1968 and #1969/#1776
pGALL-(*URA3*)-hRIPK3^VQ/AA^: #1864/#1989 and #1990/#1776

The primary PCR products to create pGALL-(*URA3*)-hRIPK3^VQVG/AAAA^ were made by amplifying pGALL-(*URA3*)-hRIPK3^VQ/AA^ with primer sets #1864/#1991 and #1776/#1992. Amplifying pGALL-(*URA3*)-hRIPK3^VQVG/AAAA^ with primer sets #1864/#1966 and #1776/#1967 produced primary PCR products for pGALL-(*URA3*)-hRIPK3^VQVG/AAAA-D142N^. Amplifying pGALL-(*URA3*)-hRIPK3 ^VQVG/AAAA^ with primer sets #1864/#1968 and #1776/#1969 yielded primary PCR products to create pGALL-(*URA3*)-hRIPK3 ^VQVG/AAAA-S227A^

pGALL-(*LEU2*)-GFP^S65T^ was made by amplifying pGALL-(*HIS3*)-GFP^S65T^ [38] with #2024 and #2025, digesting the product with BamHI and NheI and ligating into pGALL-(*LEU2*) cut with BamHI and XbaI. pGALL-(*LEU2*)-hMLKL^GFP^ was made by amplifying pGALL-(*LEU2*)-hMLKL with primers #2026 and #2027, cutting with BamHI and XbaI then ligating it into pGALL-GFP^S65T^ digested with BamHI and XbaI.

GenScript subcloned a synthetic gene (codon-optimized for expression in yeast) encoding residues 1–90 of m45 (CCE57214.1) between the BamHI and XbaI sites of pUC57. The insert was excised with BamHI and XbaI and ligated into pGALL-(*HIS3*) for yeast expression. pGALL-(*HIS3*)-BAV_Rmil was made by amplifying the cDNA from pFTRE3G PGK Puro-BAV_Rmil [39] (generously provided James Murphy) with primers #2017 and #2018, cutting with BamHI and XbaI and ligating it into pGALL-(*HIS3*) cut with BamHI and XbaI.

All insert sequences were verified by Sanger sequencing (AGRF, Melbourne, VIC, Australia). Primers used for cloning are listed in Table 1.

### 2.2. Antibodies and Reagents

Antibodies used in this study were: rat anti-hMLKL clone 3H1 [9] (kindly provided by James Murphy; commercially available as #MABC604 from Merck Millipore Burlington, MA, USA), rabbit anti-MLKL phospho S358 #AB187091 (Abcam, Cambridge, UK), Rabbit anti-hRIPK3 clone E1Z1D #13526 (Cell Signaling, Danvers, MA, USA), Rabbit anti-mRIPK3 #2283, (ProSci, Loveland, CO, USA), Mouse anti-RIPK1 # 610458 (BD Biosciences, Franklin Lakes, NJ, USA), Rabbit anti-ƴHexokinase #4959–9988, (Bio-Rad, Hercules, CA, USA), Rabbit anti-mouse IgG-HRP #A9044 (Sigma Aldrich), Donkey anti-rabbit-HRP #NA934 (Amersham, Little Chalfont, UK). Necrosulfonamide, Necrostatin-1, GSK’872 and Dabrafenib were purchased from Selleck Chemicals (Houston, TX, USA). TC13172 (HY-101524) and GW806742X (HY-112292) were purchased from MedChemExpress (Monmouth Junction, NJ, USA). DAPI was purchased from Sigma Aldrich (St. Louis, MO, USA).

### 2.3. Yeast Transformation

Yeast culturing and transformation were performed as previously described [38] except that the yeast colonies were inoculated overnight in selective repressing liquid media prior to transformation.

### 2.4. Proliferation Assays

Yeast transformants were grown in minimal-selective repressing liquid media overnight and washed three times in TE. Cell suspensions were created in 200 μL of minimal selective liquid media containing 2% *w/v* raffinose at A_620_ = 0.1 and incubated for 3 h. After incubation, 10 μL of the yeast suspensions were inoculated into either 150 μL of minimal selective repressing liquid media as uninduced controls or minimal-selective inducing liquid media (in the presence or absence of chemical inhibitors). All suspensions were cultured at 30 °C and absorbance at 620 nm was measured every 30 min for 48 h. The relative growth rates were expressed as the ratios of the maximum change in A_620_ over time of induced and uninduced yeast cultures.

### 2.5. Membrane Integrity Assays

Yeast transformants were grown in minimal selective repressing liquid media overnight, washed three times in TE, then sub-cultured into both minimal selective inducing and repressing liquid media at A_620_ = 0.1. The cells were incubated for 24 h at 30 °C. The cells were washed once in phosphate-buffered saline (PBS) and resuspended in PBS containing propidium iodide (50 μg/mL), then analyzed by flow cytometry (FACSCanto^TM^, BD Bioscience), gating on intact cells.

### 2.6. Clonogenicity

Yeast transformants were grown in minimal selective repressing liquid media overnight, washed three times in TE and resuspended in TE at A_620_ = 0.1. Twenty microliter aliquots were mixed with 2 mL TE as uninduced controls and other aliquots grown in 2 mL minimal selective inducing liquid media for 24 h. Dilutions of uninduced and induced yeast were then plated on minimal selective repressing solid media. After two days, colonies were counted and expressed relative to the colony-forming units (CFU) in the cultures prior to induction.

### 2.7. Western Blot

Yeast transformants were grown in minimal selective repressing liquid media overnight, washed three times in TE and incubated in minimal selective liquid media containing 2% (*w/v*) raffinose for 4 h, then washed once in TE and incubated in complete inducing liquid media for 7 h. After incubation, cells were pelleted and frozen before being lysed in Fastprep Lysing Matrix C 2 mL tubes (MP Biomedicals; Santa Ana, CA, USA) using a FastPrep-24 bead beating grinder and lysis system (MP Biomedicals) at a speed of 6 ms^−1^ for 40 s in cold yeast lysis buffer (20 mM Tris-HCl, 135 mM NaCl, 1.5 mM MgCl_2_, 1 mM EGTA, 10% glycerol, 1% Triton X-100, pH 7.4). Soluble lysates were boiled at 95 °C for 4 min and then resolved on 10% Tris-Glycine gels (Bio-Rad). After transfer onto PVDF membranes, membranes were blocked with 5% blocking buffer for 1 h and then probed with primary antibodies. After primary antibody incubation, the PVDF membranes were washed then incubated in HRP-conjugated secondary antibodies and washed. SuperSignal West Dura extended duration substrate (Thermo Fisher Scientific; Waltham, MA, USA) was used for detection.

### 2.8. Microscopy

Yeast transformants were grown in minimal selective repressing liquid media overnight, washed three times in TE and incubated in minimal selective liquid media containing 2% (*w/v*) raffinose for 4 h, then washed once in TE and incubated in minimal-selective inducing liquid media for 24 h. Cells were washed once in phosphate-buffered saline (PBS) and then fixed in 4% formaldehyde for 20 min and incubated in PBS containing DAPI (0.5 μg/mL) for 30 min in the dark. Cells were then washed and resuspended in PBS, mounted on coverslips 1:1 with 1% low-melting agarose and then imaged using a Zeiss 780 confocal microscope with a 63× oil immersion lens (Zeiss, Oberkochen, Germany). Scoring was performed visually in a blinded manner.

## 3. Results

### 3.1. Reconstitution of the Necrosome in Yeast

**Figure 1:**
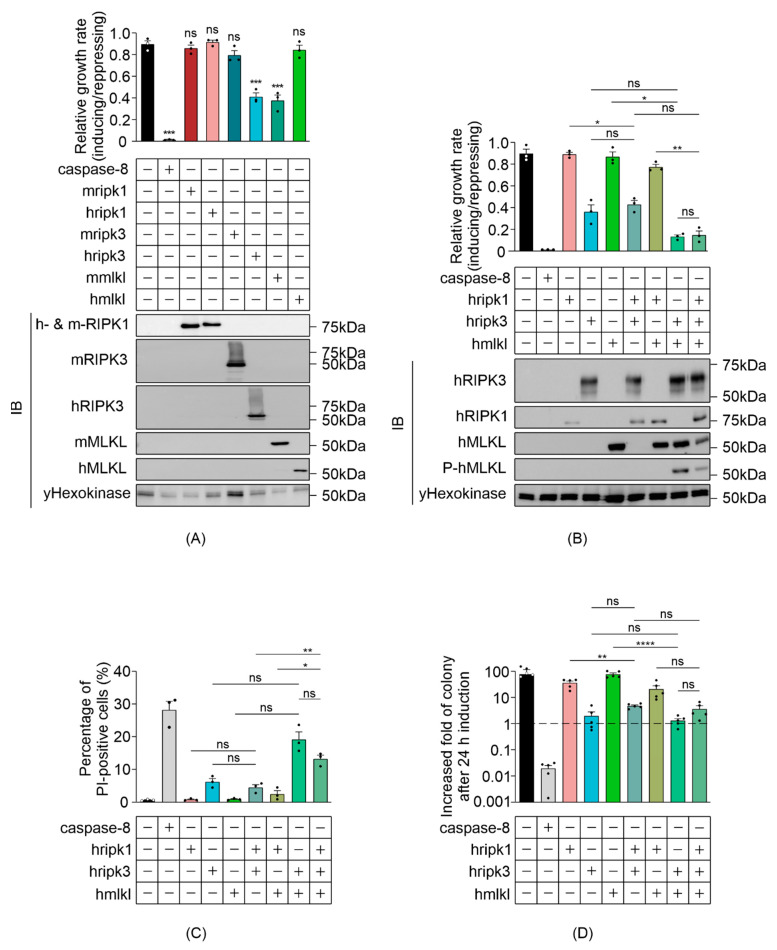
Reconstitution of necrosome interaction in *S. cerevisiae*. Maximal growth rates of yeast bearing expression plasmids encoding (**A**) single murine or human, or (**B**) human wild type necroptotic effector(s), were analyzed by monitoring changes in absorbance of yeast cultured in inducing and repressing liquid media. Expression of recombinant protein(s) were assessed by Western blot. Loading control: anti-hexokinase. (**C**) Yeast bearing expression plasmids encoding human necroptotic effector(s) were grown under inducing conditions for 24 h. Following galactose removal, plasma membrane integrity of each yeast culture was assessed by propidium iodide (PI) uptake. (**D**) The colony-forming abilities of post-induction cultures were expressed relative to the corresponding uninduced cultures. Cells were grown in inducing media for 24 h. Following galactose removal, the post-induction cultures were diluted and plated on solid repressing media and incubated for 2 days. Data present mean ± SEM of three independent assays in (**A**–**C**) and five independent assays in (**D**). Differences in growth rates of (**A**) empty vector yeast versus those expressing each transgene, or (**B**–**D**) sets of 2–3 transgenes versus individual transgenes or pairs of transgenes were compared using ANOVAs with Sidak corrections. *, *p* < 0.05; **, *p* < 0.01; ***, *p* < 0.001; ****, *p* < 0.0001; ns, not significant.

We investigated the impact of ectopic expression of necroptotic proteins in *Saccharomyces cerevisiae*, first investigating the activity of each individual component prior to exploring their interactions in the yeast context. Recent studies revealed oligomeric differences between activated mouse and human MLKL [10,11], suggesting a species-specific difference in the conformational dynamics between these necrosome orthologs. To determine the overexpression-induced activity of individual necrosome components and to examine whether human and mouse counterparts differ, we measured the maximal growth rates of yeast transformants expressing high levels of wild-type RIPK1, RIPK3 or MLKL (from human and mouse), compared with control yeast bearing empty expression vectors. Caspase-8, which was previously demonstrated to be lethal to yeast [36], acted as a positive control. Human RIPK3 and mouse MLKL significantly suppressed the growth of yeast transformants, although not as strongly as caspase-8 (Figure 1A). Consistent with a recent report [40], human RIPK1 had no significant impact on yeast growth, nor did human MLKL, mouse RIPK1 or mouse RIPK3 (Figure 1A). Western blot analysis indicated successful expression of necroptotic proteins (Figure 1A). The reduced net proliferation associated with yeast expression of murine MLKL or human RIPK3 could be a consequence of a necroptosis-like cell death, cell cycle slowing or arrest or a combination of cell death and a decrease in mitosis. The observation that yeast tolerated expression of human MLKL raised the possibility that reconstitution of the necroptotic pathway to activate human MLKL may lead to yeast growth suppression. To test this hypothesis, we analyzed the growth rate, clonogenicity and membrane integrity of yeast co-expressing wild-type human necrosome components. Human MLKL slightly suppressed the maximal growth rate of yeast co-expressing RIPK3 (Figure 1B). Co-expression of these proteins triggered membrane permeabilization in a greater proportion of yeast than the expression of RIPK3 (or hMLKL) alone (Figure 1C), but these differences were not statistically significant after adjustments for multiple comparisons. Co-expression of hMLKL had a negligible effect on the colony-forming ability of yeast transformants expressing RIPK3 (Figure 1D). These observations suggest a possible weak functional interaction between RIPK3 and MLKL in the yeast context. This notion was reinforced by the observation that co-expression of RIPK3 led to phosphorylation of serine 358 of MLKL (Figure 1B), a marker of MLKL activation [41].

We observed a non-significant trend that, relative to yeast expressing RIPK3, yeast co-expressing RIPK3 plus RIPK1 grew marginally faster, slightly fewer had permeabilized membranes, and somewhat more co-expressing cells formed colonies (Figure 1B–D). While we cannot rule out the possibility that these differences could be due to chance, the consistent trend across multiple assays hints that RIPK1 may weakly inhibit RIPK3-mediated yeast lethality, consistent with the observation from both in vitro and in vivo studies that RIPK1 inhibited receptor-independent activation of RIPK3 in mammalian cells [19,42].

The correlation between the presence of RIPK3 and MLKL phosphorylation, and the trend that co-expression of MLKL enhanced the toxicity of RIPK3 suggested that a functional RIPK3–MLKL interaction could be reconstituted in yeast. However, the ability of RIPK3 to induce yeast toxicity independent of MLKL complicated the use of this yeast model for future studies.

### 3.2. The RHIM Interacting Domain Is Not Required for MLKL Activation by RIPK3 in Yeast

**Figure 2:**
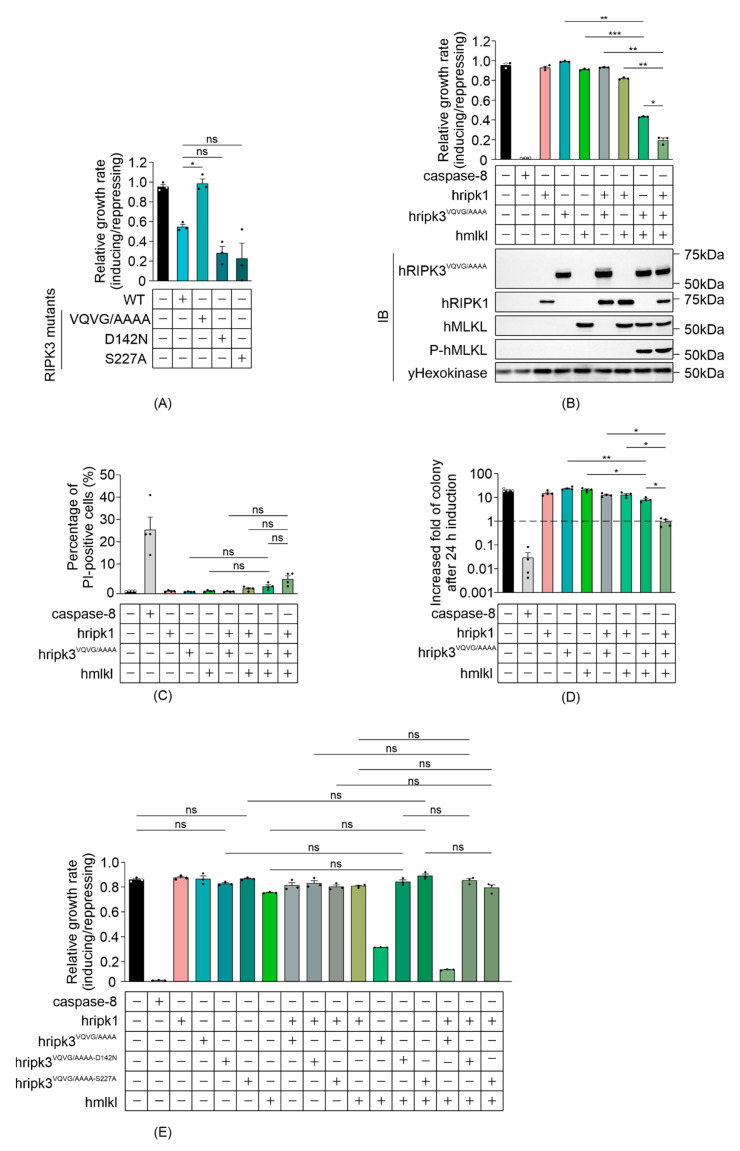
Reconstitution of necrosome interactions using RHIM-mutated RIPK3 in *S. cerevisiae*. Maximal growth rates of yeast-bearing expression plasmids encoding (**A**) human wild type and mutated RIPK3 proteins, or (**B**,**E**) human wild type MLKL, RIPK1 and/or RIPK3^VQVG/AAAA^, RIPK3^VQVG/AAAA-D142N^ or RIPK3^VQVG/AAAA-S227A^, were analyzed by monitoring changes in absorbance of yeast cultured in inducing and repressing liquid media. (**C**) Yeast bearing expression plasmids encoding human wild-type MLKL and/or RIPK1, and/or RIPK3^VQVG/AAAA^ were grown under inducing conditions for 24 h. Plasma membrane integrity of each yeast culture was assessed by propidium iodide (PI) uptake. (**D**) The clonogenic abilities of yeast transformants were evaluated by determining colony-forming ability of the culture relative before and after transgene induction for 24 h. Data present mean ± SEM of three (**A**,**B**,**E**) or four (**C**,**D**) independent assays. (**A**) Differences in growth rates of yeast expressing wild type RIPK3 and those expressing each mutated RIPK3 transgene were compared using ANOVAs with Sidak corrections. (**B**–**E**) Statistical analysis of differences in growth rates, membrane integrity and clonogenic ability between transformants expressing sets of 2–3 transgenes versus individual transgenes or pairs of transgenes were calculated using ANOVAs with Sidak corrections. *, *p* < 0.05; **, *p* < 0.01; ***, *p* < 0.001; ns, not significant.

Previous studies identified three critical factors that contribute to the function of RIPK3: catalytic activity that is essential for its function as a conventional Serine/Threonine kinase [5,43]; an intact Serine 227 residue, which is phosphorylated to allow stable binding to MLKL [39,44]; and an RHIM interaction domain, which is crucial for RHIM domain-mediated interactions with other RIPK3 monomers and distinct RHIM-containing proteins including RIPK1 [18,45]. To determine which functional domains may be responsible for the RIPK3-mediated, MLKL-independent yeast lethality, we analyzed the growth rates of yeast expressing RIPK3 variants bearing mutations in the kinase domain (RIPK3^D142N^), phosphorylation site (RIPK3^S227A^) or RHIM domain (RIPK3^VQVG/AAAA^). Mutation of the RHIM-domain completely restored the growth rate of yeast transformants, implying that the RIPK3-mediated yeast lethality was RHIM-dependent (Figure 2A). The D142N catalytically inactive mutation and the S227A phosphorylation-site mutation had no significant impact on the yeast toxicity of RIPK3 (Figure 2A). These data suggest that neither phosphorylation of residue S227 nor the catalytic activity of RIPK3 is important for yeast toxicity induced by RIPK3 in the absence of MLKL.

A previous study revealed that, although an intact RIPK3 RHIM domain was critical for the death receptor-induced necroptotic pathway, receptor-independent forced oligomerization of RHIM-mutated RIPK3 could trigger necroptosis [42]. Therefore, to define the role of RHIM-mediated interactions in the reconstitution of human necrosome pathway in yeast, the impact of a RHIM mutation was analyzed by measuring the growth of yeast co-expressing RIPK3^VQVG/AAAA^ with MLKL in the presence and absence of RIPK1. Neither RIPK3^VQVG/AAAA^ nor MLKL alone impeded yeast growth, yet co-expression dramatically reduced proliferation (Figure 2B). This indicated that the RHIM domain of RIPK3 was not critical for a functional RIPK3-MLKL interaction in yeast. The interaction was further verified by Western blot analysis: phosphorylated MLKL was detected in lysates containing RHIM-mutated RIPK3 plus MLKL (Figure 2B). These observations support prior findings using mammalian cells, demonstrating that the RHIM domain was dispensable for MLKL activation by overexpressed RIPK3 [42]. Co-expression of RIPK1 significantly enhanced RIPK3^VQVG/AAAA^/MLKL-mediated growth inhibition (Figure 2B). Taken together with the results of assays using wild type RIPK3, these data suggest that an RHIM-independent mechanism exists to facilitate RIPK3-mediated MLKL activation via RIPK1, at least in this yeast over-expression context.

We also analyzed the colony-forming ability and membrane integrity of yeast co-expressing MLKL and RHIM-mutated RIPK3, in the presence and absence of RIPK1. The plasma membranes of most of the yeast co-expressing RIPK3^VQVG/AAAA^ and MLKL remained intact after 24 h induction, whether RIPK1 was co-expressed or not (Figure 2C). While the clonogenicity of yeast co-expressing RIPK3^VQVG/AAAA^ and MLKL was partially reduced, co-expression of RIPK1 with those proteins for 24 h prevented any net increase in colony-forming cells (Figure 2D). These results suggest that in yeast, RIPK1 facilitated MLKL activation by activating RIPK3^VQVG/AAAA^, reducing proliferation.

We tested the importance of the catalytic activity of RIPK3 and its S227 phosphorylation site for MLKL phosphorylation in yeast, in the context of a RIPK3 protein bearing a mutated RHIM domain. MLKL failed to cooperate with RIPK3 proteins bearing an RHIM mutation coupled with mutations in either the kinase domain or phosphorylation site (RIPK3^VQVG/AAAA-D142N^ or RIPK3^VQVG/AAAA-S227A^) to impede yeast growth, regardless of RIPK1 presence or absence (Figure 2E). These results indicated that both the catalytic activity of RIPK3 and an intact S227 phosphorylation site were crucial for RIPK3 to functionally interact with MLKL in yeast, mirroring RIPK3-MLKL interactions in mammalian cells.

### 3.3. RIPK3 Induces Aggregation of MLKL in Yeast

**Figure 3:**
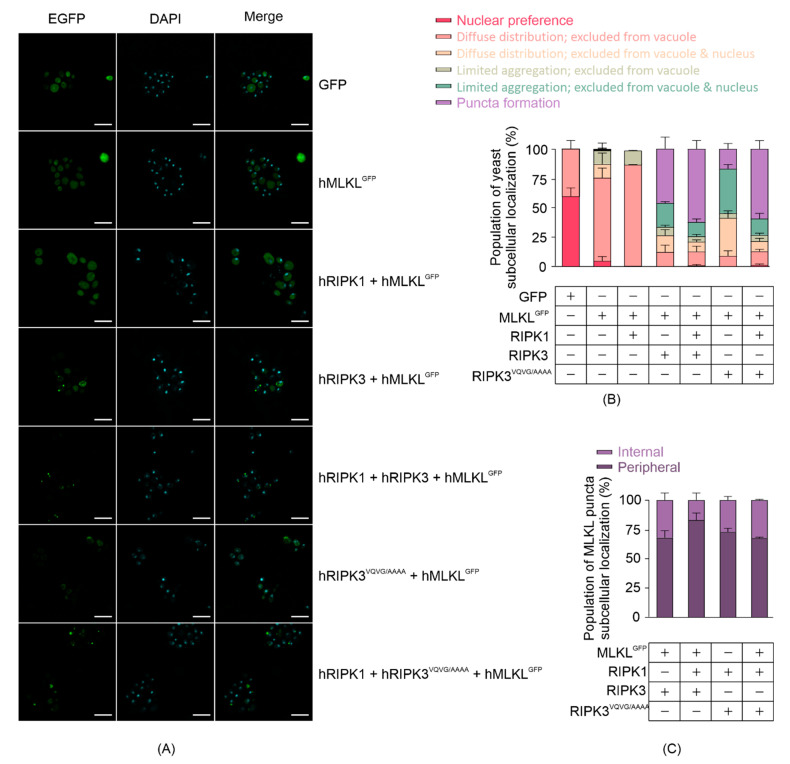
MLKL activation leads to its aggregation in yeast. (**A**) Subcellular distribution of C-terminal GFP-tagged MLKL in yeast after 24 h-induction in liquid media. Scale bars are 10 µm. Quantitative analyses of (**B**) yeast populations in terms of the subcellular distribution of GFP-tagged MLKL, and (**C**) subcellular localization of MLKL^GFP^ puncta in yeast were assessed by confocal microscopy. Yeast expressing GFP or GFP-tagged MLKL with or without RIPK1 and/or RIPK3 were grown in inducing media for 24 h. At least 35 cells per yeast culture were counted in each independent assay. Graphs present mean ± SEM of three independent assays.

Existing literature illustrates that active MLKL interacts directly with the plasma membrane by binding with phospholipids. To assess MLKL subcellular localization in yeast, we analyzed yeast expressing carboxyl-terminally GFP-tagged human MLKL 24 h post-induction using confocal microscopy (Figure 3A). We categorized yeast cells into six classes in terms of GFP or MLKL^GFP^ distribution: 1. preferentially nuclear; 2. diffuse distribution but excluded from the vacuole; 3. diffuse distribution but excluded from the vacuole and nucleus; 4. limited aggregation and excluded from the vacuole; 5. limited aggregation and excluded from the vacuole and nucleus; 6. puncta formation. Most MLKL^GFP^ was diffusely distributed but excluded from the vacuole in yeast when expressed without other necrosome components or when co-expressed with RIPK1 (Figure 3B). In the presence of RIPK3 or RIPK3^VQVG/AAAA^, however, the majority of MLKL^GFP^ was excluded from the nucleus and tended to aggregate or form puncta (Figure 3B). Most MLKL^GFP^ puncta were observed in the periphery of the cells, consistent with membrane localization (Figure 3C). The observation that MLKL formed puncta and became phosphorylated in yeast when co-expressed with RIPK3 is consistent with findings from other researchers using mammalian cells, showing that MLKL aggregated into large molecular weight complexes upon activation [24].

### 3.4. Some MLKL and RIPK1 Chemical Inhibitors Interfere with Necrosome Function in Yeast

**Figure 4:**
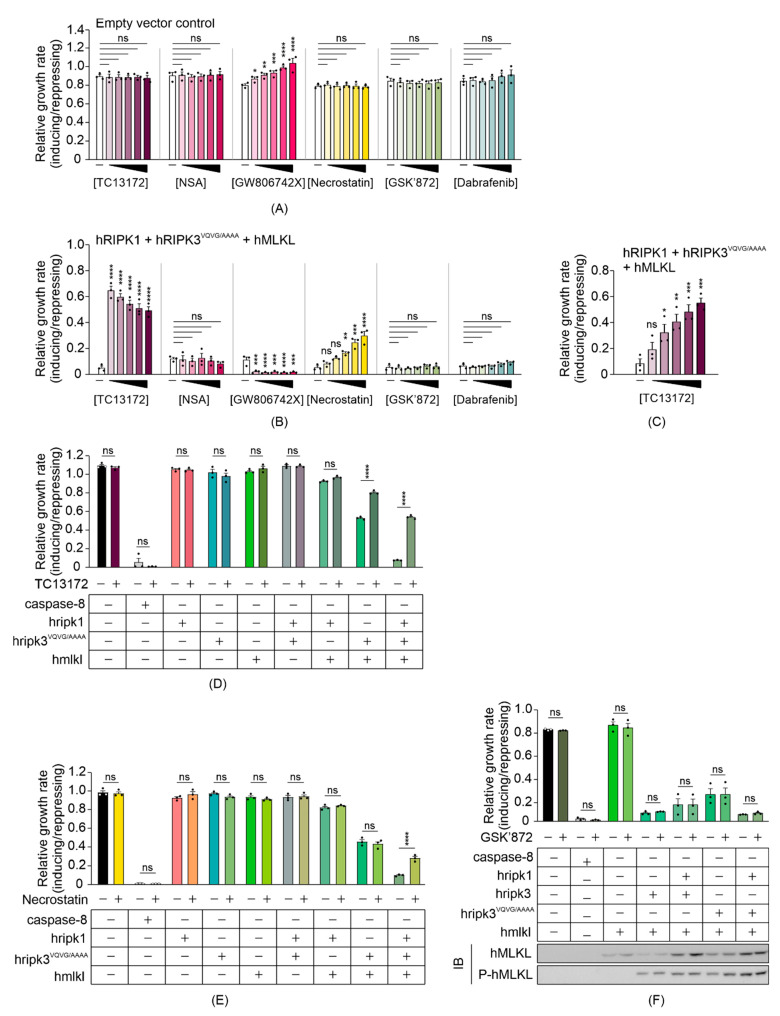
TC13172 and necrostatin inhibit necrosome interaction-induced yeast lethality. Maximal growth rates of yeast bearing (**A**) empty vectors or (**B**,**C**) expression plasmids encoding human wild-type MLKL, RIPK1 and RIPK3^VQVG/AAAA^ in inducing versus repressing liquid media with different concentrations of indicated small molecule necroptotic inhibitors. Differences in growth rates of yeast in media either without drugs (-) or containing (**A**,**B**) 20, 40, 60, 80 or 100 µM of TC13172, NSA, GW806742X, necrostatin, GSK’872 or Dabrafenib; or (**C**) 4, 8, 12, 16 or 20 µM of TC13172 (increasing concentrations indicated by black triangle) were compared. Graphs present mean ± SEM of three independent assays. The effects of (**D**) 20 µM TC13172, (**E**) 100 µM necrostatin or (**F**) 100 µM GSK’872 on the growth rates of yeast expressing either MLKL, RIPK3, RIPK3^VQVG/AAAA^ or RIPK1 or co-expression of two or three transgenes were assessed by monitoring the change in absorbance of yeast cultures in inducing and repressing media. The effect of GSK’872 on proliferation and MLKL phosphorylation of yeast bearing various transgenes were assayed (**F**). Differences in growth rates of untreated versus drug-treated yeast were compared using ANOVAs with Sidak corrections (**A**–**F**). Graphs present mean ± SEM of three independent assays. *, *p* < 0.05; **, *p* < 0.01; ***, *p* < 0.001; ****, *p* < 0.0001; ns, not significant.

Compared to mammalian cells, our yeast model provides a naïve system that may be useful for screening for chemicals or proteins that target the necroptotic signaling pathway. Since expressing functional necrosome complexes suppressed the maximum growth rate of yeast transformants, we expected necroptotic inhibitors would restore the growth by interfering with the activity of, or interactions between, the co-expressed necrosome components. To determine the feasibility of the yeast model in screening for potential necrosome inhibitors, we tested the impact of some small-molecule necroptotic inhibitors on the growth of yeast co-expressing necrosome components. Necrosulfonamide (NSA) [44] and TC13172 [46] are MLKL inhibitors that target the C86 residue of human MLKL, preventing MLKL oligomerization and thus inhibiting necroptosis. GW806742X is an MLKL inhibitor that binds to the pseudokinase domain, preventing membrane translocation of MLKL [10]. Dabrafenib [47] and GSK’872 [48] are RIPK3 kinase inhibitors, and necrostatin allosterically inhibits RIPK1, preventing its pro-necroptotic interaction with RIPK3 [49]. Since all of the inhibitors were dissolved in DMSO, we first tested the impact of DMSO on the growth of yeast cells in both repressing and inducing liquid media at concentrations up to 5%. DMSO showed a concentration-dependent toxicity to yeast bearing empty vectors, but 1% DMSO was well tolerated (Figure S1A). Therefore, to minimize solvent effects, we tested the candidate inhibitors on yeast at concentrations up to 100 µM in liquid-inducing or -repressing media containing a total of 1% DMSO. The non-specific effects of each inhibitor were first tested against yeast bearing empty vectors. NSA, necrostatin, GSK’872 and dabrafenib did not affect the growth of yeast bearing empty vectors (Figure 4A). GW806742X did modulate proliferation of the control yeast, however. It slowed the proliferation of control yeast to a greater extent in repressing (glucose-containing) media than in inducing (galactose-containing) media (Figure S1B,C), accounting for the apparent increase in relative growth rate when presented as the ratio of proliferation in inducing versus repressing media in Figure 4A. This compound was documented to target multiple mammalian kinases [46,50] so we attribute its effect on the growth of yeast lacking necroptotic transgenes to inhibition of yeast kinases.

Exposure to 20 µM TC13172 nearly completely restored the maximum growth rate of yeast expressing RIPK1, RIPK3^VQVG/AAAA^ and MLKL, increasing proliferative rate by approximately 22-fold relative to the untreated yeast expressing those proteins; however, concentration-dependent toxicity of TC13172 was observed when the necroptotic pathway components were induced (Figure 4B). At lower concentrations, a dose-dependent increase in proliferation was observed (Figure 4C). Necrostatin also boosted the maximum growth rate of yeast transformants expressing RIPK1, RIPK3^VQVG/AAAA^ and MLKL in a concentration-dependent manner up to 100 µM (Figure 4B). Neither GSK’872 nor dabrafenib affected the proliferation of yeast expressing the necroptotic effectors. Instead of the expected inhibition of MLKL-dependent suppression of yeast growth, GW806742X toxicity was observed (Figure 4B).

To define the molecular targets of the functional small molecule inhibitors in the yeast model, we treated yeast expressing various necrosome components with TC13172 or necrostatin at their most effective concentrations. Treatment with 20 µM of TC13172 did not affect the anti-proliferative effect of caspase-8 expression, but significantly enhanced the growth rate of yeast expressing functional necrosome complexes containing active MLKL, which is consistent with TC13172-suppressing MLKL activity in yeast (Figure 4D).

Treatment with 100 µM of necrostatin failed to protect yeast from the toxic effects of caspase-8 or co-expression of RIPK3^VQVG/AAAA^ plus MLKL but significantly impaired the ability of RIPK1 to exacerbate the toxicity associated with RIPK3^VQVG/AAAA^/MLKL co-expression, confirming that necrostatin could diminish necrosome-mediated yeast toxicity by inhibiting RIPK1 activity (Figure 4E).

Interestingly, the chemical inhibitors targeting the kinase activity of RIPK3 did not suppress RIPK1/RIPK3^VQVG/AAAA^/MLKL-mediated yeast toxicity (Figure 4B). This observation contrasted with the impact of mutating the kinase domain of RIPK3, which completely neutralized this yeast toxicity (Figure 2E). Treating yeast transformants with 100 µM GSK’872 did not affect the growth suppression associated with wild-type or RHIM-mutated RIPK3, with or without MLKL and/or RIPK1 and also failed to impact MLKL phosphorylation by RIPK3 (Figure 4F). This observation indicated that GSK’872 did not impact the catalytic activity of RIPK3 in the yeast context.

These data reveal that yeast reconstituted with a necroptotic pathway culminating in MLKL activation could model the activities of a subset of small molecule inhibitors targeting RIPK1 and MLKL.

### 3.5. MLKL and RIPK3 Viral Inhibitors Interfere with Necrosome Function in Yeast

**Figure 5:**
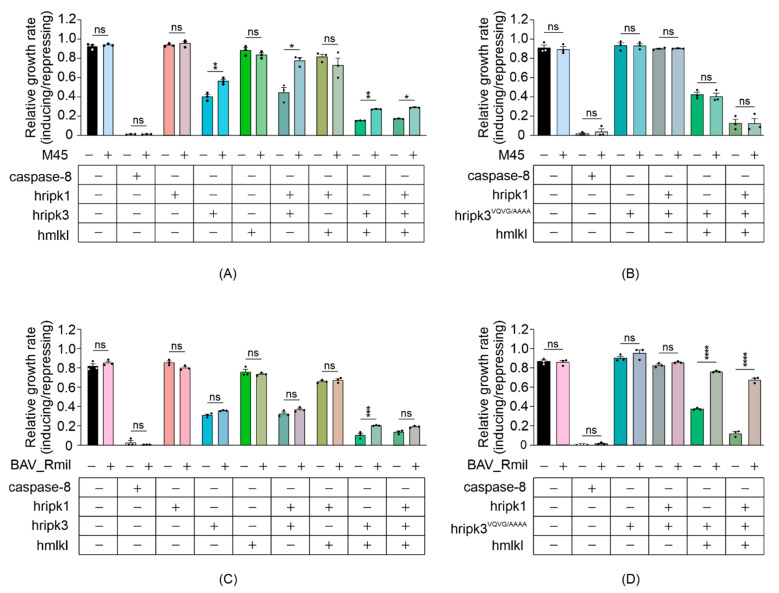
Viral necroptotic inhibitors reduce necrosome reconstitution-induced yeast lethality. (**A**,**B**) The effects of M45 on the growth of yeast in liquid media were analyzed by co-expressing necrosome effector(s) in yeast. Maximal growth rate of yeast bearing expression plasmids encoding human wild-type MLKL, RIPK1 and either RIPK3 (**A**,**C**), or RIPK3^VQVG/AAAA^ (**B**,**D**), with and without M45 (**A**,**B**) or BAV_Rmil (**C**,**D**) in liquid media, were analyzed by monitoring the relative maximal changes in absorbance between yeast cultures in inducing and repressing media. Differences in growth rates of yeast expressing necrosome effectors with or without the viral inhibitors were compared using ANOVAs with Sidak corrections. Data present mean ± SEM of three independent assays. *, *p* < 0.05; **, *p* < 0.01; ***, *p* < 0.001; ****, *p* < 0.0001 ns, not significant.

Other than caspase-8, which can cleave RIPK1 [17] (and possibly RIPK3 [51]) to preclude their activation of MLKL in mammalian cells, the only necroptotic inhibitory proteins that have been identified to date are viral proteins that act through dominant negative mechanisms. To determine the utility of yeast bearing necroptotic regulators for identifying and characterizing protein inhibitors of this pathway, we expressed two viral necroptosis inhibitors in yeast reconstituted with necrosome components: cytomegalovirus M45 and poxvirus BAV_Rmil. Previous work revealed that M45 and RIPK3 interact via their RHIM domains, and this association prevents RIPK3 from being activated by RIPK1 [52]. BAV_Rmil competes with MLKL for binding to RIPK3 and thus inhibits RIPK3-mediated MLKL activation [39]. Co-expressing M45 with wild-type RIPK3-containing necrosomes boosted the maximum growth rate of yeast transformants (Figure 5A). Unsurprisingly, yeast lethality mediated by RIPK3^RHIM^-containing functional necrosomes was immune to co-expression of M45 (Figure 5B). Co-expression of BAV_Rmil afforded slight protection against RIPK3-MLKL-induced yeast growth suppression (Figure 5C); however, it dramatically reversed the yeast growth suppression mediated by RIPK3^VQVG/AAAA^ plus MLKL in the presence or absence of RIPK1 (Figure 5D). These data illustrate the applicability of this yeast model for screening for endogenous or pathogen-encoded necroptotic inhibitors and investigations into their functions and specificities.

## 4. Discussion

Overexpression of many mammalian proteins has been noted to suppress the proliferation of yeast cells or induce their death. These include proteases including caspases [38], mitochondrial proteins like Bax [38], many kinases [40], transcription factors including p53 [53], the PAR polymerizing enzymes PARP 1 and 2 [54] and amyloidogenic proteins [55]. Scientists have capitalized on these observations to reconstitute and interrogate pathways culminating in activation of these proteins and to identify and characterize protein and chemical modulators of those pathways [38]. We reasoned that the ability of active MLKL to permeabilize cell membranes may render it toxic to yeast as well as mammalian cells. We hoped that such toxicity may facilitate reconstitution of the core necroptotic pathway in yeast, providing a naïve eukaryotic platform for researching factors that regulate necroptosis.

Endogenous necrosome complex assembly requires activation of either death receptors, cytokine receptors or immune receptors via ligand binding [3,4]. Once activated, the ligand–receptor complex recruits RIPK1, which in turn initiates the formation of necrosome by recruiting RIPK3. In some circumstances, RIPK1 is dispensable when the ligand-receptor complex directly forms an RHIM-dependent complex with RIPK3 [48,56]. Within necrosomes, RIPK3 recruits and activates MLKL, which oligomerizes and translocates to the plasma membrane to compromise its integrity [20,21,22]. Here we present reconstitution of mammalian necrosomes in yeast, utilizing overexpression to activate the components in a stimulus-independent manner.

Our data supported the findings from mammalian cells that differences exist between human and mouse necrosomes at the molecular level. While human MLKL needed to be activated to induce yeast cell death, mere expression of mouse MLKL dramatically slowed yeast proliferation, implying that human MLKL folds in a relatively more stable inactive conformation than mouse MLKL in yeast, as in mammalian cells [57,58].

Although the functional link between RIPK3 expression and phosphorylated MLKL mirrored the interaction between RIPK3 and MLKL within necrosomes in human cells, where activated RIPK3 binds to and phosphorylates MLKL, the spontaneous yeast toxicity of wild-type RIPK3 complicated our attempts to model MLKL-mediated cell death. The simplest explanation for the toxicity of RIPK3 in yeast is that high-level expression of an active kinase led to phosphorylation-mediated dysregulation of the activity or interactions of essential yeast proteins. Indeed, about 5% of human kinases were recently reported to compromise yeast viability upon overexpression [40]. However, wild-type and RHIM-mutated RIPK3 phosphorylated MLKL to similar extents, suggesting their kinase activities may be comparable, yet only wild-type RIPK3 was toxic to yeast. Furthermore, the kinase-dead D142N mutant of RIPK3 was lethal to yeast. As RIPK3 has been shown to form amyloid fibrils in vitro in an RHIM-dependent manner [18] and other amyloid-forming proteins have been reported to kill yeast [55], we conclude that the yeast toxicity triggered by overexpression of wild-type RIPK3 probably results from RHIM-mediated fibril formation.

Our finding that a RIPK3 mutant lacking an intact RHIM domain was capable of phosphorylating and activating MLKL contradicted some published literature suggesting that the RHIM domain of RIPK3 was essential for necroptosis [5] and indicated that (in yeast at least) RIPK3 can activate MLKL in an RHIM-independent manner. This was reminiscent of the outcome of forced oligomerization of RHIM domain-mutated RIPK3 under receptor-independent conditions in mammalian cells [6]. We suspect that the strong galactose-inducible transgene expression [38] achieved in this study would produce much higher intracellular concentrations of RIPK3 and MLKL than would result from endogenous expression in human cells. The elevated concentration possibly overcame an endogenous requirement for an intact RHIM domain to mediate the RIPK3 dimerization needed to achieve MLKL activation. Fortuitously, mutation of the RHIM domain of RIPK3 neutralized its toxicity, enabling reconstitution of a necroptotic pathway in yeast that models RIPK1-facilitated, RIPK3-dependent MLKL activation.

While RIPK1 exacerbated the yeast toxicity provoked by co-expression of RHIM-mutated RIPK3 plus MLKL, it failed to accentuate the yeast lethality seen when wild-type RIPK3 was expressed (with or without MLKL). Indeed, RIPK1 seemed slightly protective in that context. In mice, RIPK1 was shown to counteract ZBP-1/RIPK3-mediated necroptosis by interfering with the RHIM domain-mediated interaction [59], implying that RIPK1 could competitively bind with RIPK3 via its RHIM domain. It is possible that the tendency of RIPK1 to dampen the yeast toxicity induced by wild-type RIPK3 reflects an ability of the RIPK1 RHIM domain to prevent productive RHIM:RHIM interactions within RIPK3 proteins.

Propidium iodide uptake assays confirmed that at least some of the growth suppression mediated by active MLKL was due to the death of the yeast. By comparing the subcellular localization of GFP-tagged MLKL in its inactive or active state, confocal microscopy showed that co-expression of RIPK3 induced MLKL export from nuclei and aggregation in the periphery of the cell. This is consistent with existing literature revealing that inactive MLKL is a nucleo-cytoplasmic shuttling protein, whose phosphorylation by RIPK3 initially induces nuclear import and low-order oligomerization and then subsequent cytosolic export and higher-order oligomerization [60]. Considered with our data that some yeast co-expressing MLKL and RIPK3 have damaged membranes, we infer that the puncta containing MLKL formed pores in the yeast plasma membrane.

M45, a viral MLKL inhibitor that incapacitates RIPK3 via an RHIM-mediated dominant negative mechanism, partially restored the proliferation of yeast expressing wild-type RIPK3, confirming that the yeast toxicity associated with wild-type RIPK3 involved RHIM-mediated interactions, probably with other RIPK3 monomers but possibly also with yeast proteins. Mutation of the RIPK3 RHIM domain mutation nullified MLKL-independent RIPK3 toxicity, but the mutant RIPK3 retained the ability to functionally interact with necrosome components in the yeast context. The context-dependent activities of necrostatin, TC13172 and BAV_Rmil in yeast confirmed the RHIM-independent RIPK1-RIPK3 interaction, RIPK3-MLKL interaction and MLKL oligomerization, respectively.

Although TC13172 worked as expected in the yeast model, the other MLKL inhibitors NSA and GW806742X were ineffective. TC13172 was reportedly approximately 200–300 times more potent than NSA or GW806724 at blocking necroptotic mammalian cell death [46], so our system is probably not sensitive enough to discern the activity of those less potent drugs. The RIPK3 kinase inhibitors (Dabrafenib and GSK’872) we tested also failed to restore the growth of yeast expressing lethal necrosome complexes. This was surprising, given that necrostatin was active in yeast and its published IC_50_ for RIPK1 inhibition [61] was about 8-fold or 1500-fold higher than the IC_50_ values for RIPK3 inhibition by dabrafenib [47] or GSK’872 [62], respectively. The inability of GSK’872 to protect yeast from necrosome-mediated lethality correlated with its inability to prevent RIPK3-mediated phosphorylation of MLKL. This lack of activity could be due to two possible reasons: 1. The drugs may have failed to penetrate through the yeast cell wall and/or plasma membrane or were rapidly pumped out; or 2. RIPK3 may not be susceptible to these agents in the yeast context, perhaps due to altered folding or post-translational modifications. Our data do not allow us to evaluate the second possibility, but the first does seem a plausible explanation (for the inactivity of Dabrafenib at least). An in vivo study focusing on treatment of melanoma brain metastases using dabrafenib revealed that P-glycoprotein and breast cancer resistance protein efficiently pump dabrafenib to the extracellular matrix [63]. Since P-glycoprotein belongs to an ATP-binding cassette (ABC) sub-family [64], we speculate that yeast ABC family proteins, such as PDR-encoded multidrug resistance transporter [65,66], may pump dabrafenib out of yeast cells, limiting its intracellular drug concentration in yeast. We are not aware of studies investigating whether membrane transporters can pump GSK’872 out of cells, so we cannot speculate on the likelihood that efflux of GSK’872 from yeast accounts for its lack of activity in this model. Although not all candidate necroptotic inhibitors were compatible with our yeast model, it provides a naïve platform for screening for potential inhibitors.

In this study, we reconstituted necrosome complexes in yeast, confirming previously observed species-specific differences between murine and human MLKL orthologs. This yeast model provides a potentially high-throughput tool to investigate mechanisms regulating necroptosis and to screen for or characterize small molecule or protein inhibitors targeting specific necrosome components.

## Figures and Tables

**Table 1:** Primers used to generate plasmids via PCR.

Target Plasmid	Primer Sequence 5′–3′	Template	Number
pGALL-(*LEU2*)-hMLKL	AGAAAAAACCCCGGATCCATGGAAAATTTGAAGCATATTATCACC	Human *mlkl*	#1
	AAGCAGAGATTATCTAGACTACTTAGAAAAGGTGGAGAGTTTCT		#2
	TCTAGATAATCTCTGCTTTTGTGCG	pGALL-(*LEU2*)	#3
	GGATCCGGGGTTTTTTCTCCTTG		#4
pGALL-(*TRP1*)-hRIPK1	TTTGAATTCATGCAACCAGACATGTCCT	Human *ripk1*	#2014
	TTTCTCGAGTTAGTTCTGGCTGACGTAA		#2015
pGALL-(*URA3*)-hRIPK3^D142N^	CCACTTTAACTAATACTTTCAACATTTTCGG	pGALL-(*URA3*)-hRIPK3	#1864
	ATGGCTTCAAATTTCTATGCAACAA		#1966
	TTGTTGCATAGAAATTTGAAGCCAT		#1967
	CTTTATTATTTTTATTTTATTGAGAGGGTGG		#1776
pGALL-(*URA3*)-hRIPK3^S227A^	CCACTTTAACTAATACTTTCAACATTTTCGG	pGALL-(*URA3*)-hRIPK3	#1864
	CGTAAACTAAAGCTGGTTCAGTTGG		#1968
	CCAACTGAACCAGCTTTAGTTTACG		#1969
	CTTTATTATTTTTATTTTATTGAGAGGGTGG		#1776
pGALL-(*URA3*)-hRIPK3^VQ/AA^	CCACTTTAACTAATACTTTCAACATTTTCGG	pGALL-(*URA3*)-hRIPK3	#1864
	CACCAACAGCAGCACCAGA		#1989
	TCTGGTGCTGCTGTTGGTG		#1990
	CTTTATTATTTTTATTTTATTGAGAGGGTGG		#1776
pGALL-(*URA3*)-hRIPK3^VQVG/AAAA^	CCACTTTAACTAATACTTTCAACATTTTCGG	pGALL-(*URA3*)-hRIPK3^VQ/AA^	#1864
	GTTGTCAGCAGCAGCAGC		#1991
	GCTGCTGCTGCTGACAAC		#1992
	CTTTATTATTTTTATTTTATTGAGAGGGTGG		#1776
pGALL-(*LEU2*)-GFP^S65T^	GCTGGATCCGCCTCTAGAATGGGTAAAGGAGAAGAAC	pGALL-(*HIS3*)-GFP^S65T^	#2024
	TTCTTGCTAGCTTATTTGTATAGTTCATC		#2025
pGALL-(*LEU2*)-hMLKL^GFP^	GCTGGATCCATGGAAAATTTGAAGCATATT	pGALL-(*LEU2*)-hMLKL	#2026
	GCCTCTAGACTTAGAAAAGGTGGAGAGTTT		#2027
pGALL-(*HIS3*)-BAV_Rmil	GCGGATCCACCATGACTGATCCCCTGTTGCACAA	pFTRE3G PGK Puro-BAV_Rmil	#2017
	TTTTCTAGATTACTCAATCTTCTTGTTGAACTTGTAGATT		#2018

## Data Availability

The authors confirm that the data supporting the findings of this study are available in the manuscript.

## Supplementary Materials

The following are available online at https://www.mdpi.com/2218-273X/11/2/153/s1, Figure S1: Determination of DMSO and GW806742X-mediated yeast toxicity.
