# Supplementary material for: Reconstitution of Human Necrosome Interactions in Saccharomyces cerevisiae
Source: Biomolecules. 2021 Jan 25;11(2):153. doi: 10.3390/biom11020153 (PMC7911209; doi:10.3390/biom11020153)
Supplement: Supplementary file 1 [file biomolecules-11-00153-s001.pdf]

biomolecules

Supplementary materials

Reconstitution of Human Necrosome Interactions in Saccharomyces Cerevisiae

Y. Ji, L. A. Ward and C. J. Hawkins *

Department of Biochemistry and Genetics, La Trobe Institute for Molecular Science, La Trobe University, Bundoora, VIC, Australia; Y.Ji@latrobe.edu.au (Y.J.); L.Ward@latrobe.edu.au (L.A.W.)
* Correspondence: c.hawkins@latrobe.edu.au

Citation: Y. Ji; L. A. Ward; C. J. Hawkins Reconstitution of Human Necrosome Interactions in Saccharomyces Cerevisiae. **2021**, *10*, 153. https://doi.org/10.3390/biom11020153

Received: 07 December 2020
Accepted: 20 January 2021
Published: date

Publisher's Note: MDPI stays neutral with regard to jurisdictional claims in published maps and institutional affiliations.

Copyright: © 2021 by the authors. Submitted for possible open access publication under the terms and conditions of the Creative Commons Attribution (CC BY) license (http://creativecommons.org/licenses/by/4.0/).

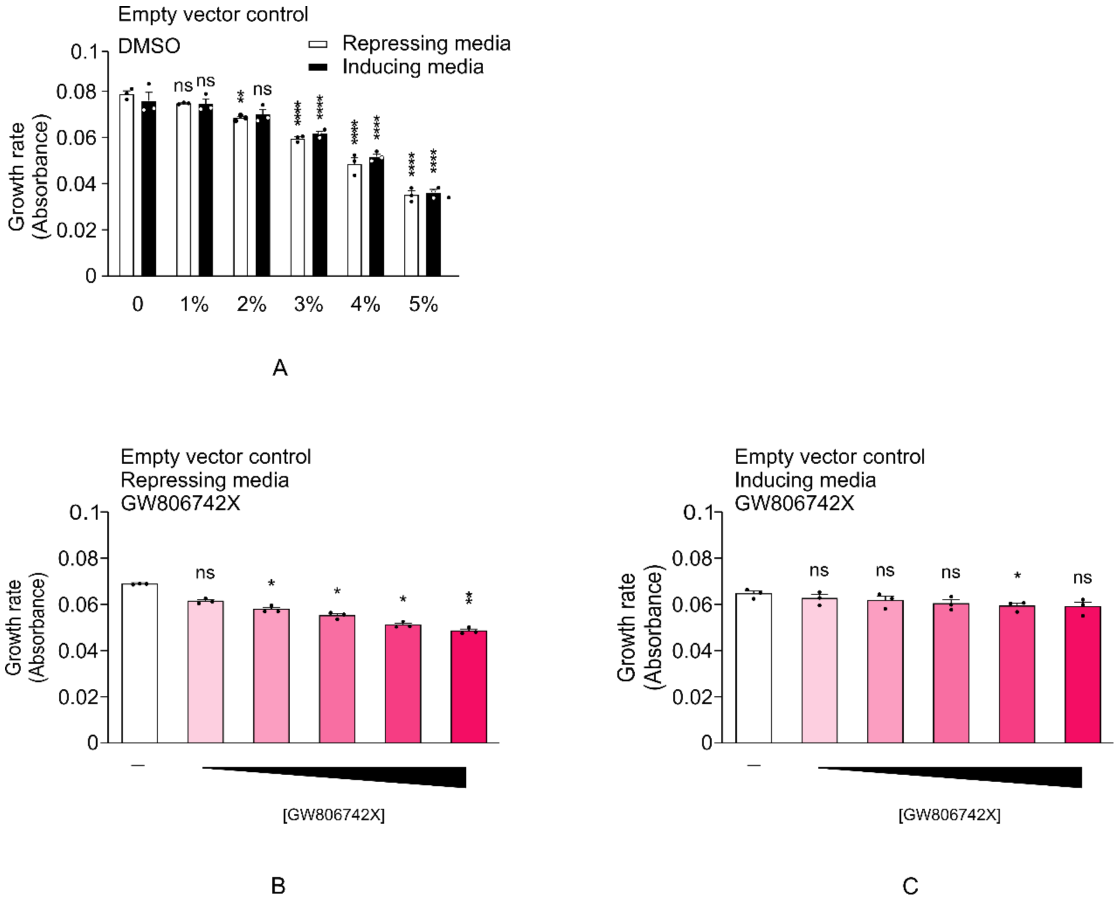

Figure S1. Determination of DMSO and GW806742X-mediated yeast toxicity. (A) The inhibitory effects of DMSO on the growth of yeast in liquid media were analyzed by measuring the change in absorbance of yeast cultures in inducing or repressing media with different concentrations of DMSO. Differences in growth rates of empty vector yeast in media without DMSO and those expressing in liquid media the indicated concentrations of DMSO were compared using ANOVAs with Sidak corrections. Maximal growth rate of yeast bearing empty vectors were measured by absorbance of yeast culture grown in inducing (B) or repressing (C) media with different concentrations of GW806742X. Differences in growth rates of yeast in media either without drugs (-) or containing 20, 40, 60, 80 or 100 μM of GW806742X were compared using ANOVAs with Sidak corrections. Data present mean ± SEM of three independent assays. Statistical analysis of differences in growth rates were indicated in the figures. *, $P < 0.05$; **, $P < 0.01$; ***, $P < 0.001$; ****, $P < 0.0001$; ns, not significant..
